# Application of artificial neural network to investigate the effects of 5-fluorouracil on ribonucleotides and deoxyribonucleotides in HepG2 cells

**DOI:** 10.1038/srep16861

**Published:** 2015-11-18

**Authors:** Jianru Guo, QianQian Chen, Christopher Wai Kei Lam, Caiyun Wang, Vincent Kam Wai Wong, Fengguo Xu, ZhiHong Jiang, Wei Zhang

**Affiliations:** 1 State Key Laboratory of Quality Research in Chinese Medicine, Macau Institute for Applied Research in Medicine and Health, Macau University of Science and Technology, Taipa, Macau, China; 2 Key Laboratory of Drug Quality Control and Pharmacovigilance (Ministry of Education), China Pharmaceutical University, Nanjing, 210009, China

## Abstract

Endogenous ribonucleotides and deoxyribonucleotides are essential metabolites that play important roles in a broad range of key cellular functions. Their intracellular levels could also reflect the action of nucleoside analogues. We investigated the effects of 5-fluorouracil (5-FU) on ribonucleotide and deoxyribonucleotide pool sizes in cells upon exposure to 5-FU for different durations. Unsupervised and supervised artificial neural networks were compared for comprehensive analysis of global responses to 5-FU. As expected, deoxyuridine monophosphate (dUMP) increased after 5-FU incubation due to the inhibition of thymine monophosphate (TMP) synthesis. Interestingly, the accumulation of dUMP could not lead to increased levels of deoxyuridine triphosphate (dUTP) and deoxyuridine diphosphate (dUDP). After the initial fall in intracellular deoxythymidine triphosphate (TTP) concentration, its level recovered and increased from 48 h exposure to 5-FU, although deoxythymidine diphosphate (TDP) and TMP continued to decrease compared with the control group. These findings suggest 5-FU treatment caused unexpected changes in intracellular purine polls, such as increases in deoxyadenosine triphosphate (dATP), adenosine-triphosphate (ATP), guanosine triphosphate (GTP) pools. Further elucidation of the mechanism of action of 5-FU in causing these changes should enhance development of strategies that will increase the anticancer activity of 5-FU while decreasing its resistance.

The ribonucleotides (RN) and deoxyribonucleotides (dRN) are essential metabolites that play important roles in a broad range of key cellular functions such as DNA synthesis and repair as well as energy metabolism^1^,^2^,^3^,^4^. Deoxyribouncleoside monophosphates (dNMP), deoxyribouncleoside diphosphates (dNDP) and deoxyribouncleoside triphosphates (dNTP) are major metabolites of dRN metabolism and building blocks of DNA synthesis^5^. Correspondingly, ribouncleoside monophosphates (NMP), ribouncleoside diphosphates (NDP) and ribouncleoside triphosphates (NTP) are major metabolites of RN metabolism^6^. Factors affecting RN and dRN pool sizes could alter cellular functions.

Nucleoside analogues are used as anticancer and antiviral drugs^7^,^8^,^9^,^10^. They undergo a stepwise intracellular phosphorylation to their triphosphate metabolites, which are preferentially incorporated into growing DNA to cause premature chain termination or inhibition of key enzymes^11^,^12^,^13^,^14^. The action of nucleoside analogues against cancer and viral infection could be affected by RN and dRN pool sizes^11^,^15^,^16^,^17^. Until recently, methods for assessment of RN and dRN pool sizes were not available. We have developed such method to study the perturbation of RN and dRN in cancer cell lines incubated with hydroxyurea and aphidicolin^18^.

5-Fluorouracil (5-FU) is one of the most commonly used anti-cancer drugs that has been used to treat many types of cancers such as breast, colorectal cancer, esophageal, and stomach cancers^19^,^20^,^21^. It is metabolized in cells to RN and dRN that result in both DNA-directed and RNA-directed cytotoxicites^22^,^23^,^24^. Its active metabolite, 5-fluoroxyuridine triphosphate (FUTP), corporates extensively into RNA strands thereby inhibiting RNA synthesis and stopping the growth of cancerous cells. Another active metabolite, 5-fluoro-2′-deoxyuridine-5′-monophosphate (FdUMP), inhibits the action of thymidylate synthase (TS), which is responsible for the conversion of dUMP to TMP. FdUMP binds to the nucleotide binding site of TS and blocks the binding of the normal substrate dUMP leading to inhibition of TMP synthesis^25^,^26^. TS inhibition results in accumulation of dUMP and depletion of deoxythymidine metabolites. A previous study has observed that 5-FU incubation of human colon carcinoma cells resulted in decrease of TTP and increase of dATP concentration without effect on deoxyguanosine triphosphate (dGTP) and deoxycytidine triphosphate (dCTP) concentration^27^. Others found lower levels of TTP and elevated of dGTP, dATP and dCTP when mouse 5178Y lymphoma cells was incubated with different concentrations of 5-FU^28^. It was also reported that 5-FU incubation resulted in increased dUMP/TMP ratio in budding yeast^29^.

However, there has been no report on the effects of 5-FU incubation on RN and dRN pool sizes due mainly to the difficulty of quantifying these pool sizes intracellularly. Therefore, the exact mechanism of 5-FU’s anticancer activity has not been fully elucidated. In the present study, we investigated the effects of 5-FU incubation over different time-periods on RN and dRN pool sizes of a human hepatocarcinonma cancer (HepG2) cell line using HPLC/MS/MS methodology.

Artificial neural network methods are an efficient technology to categorize RN and dRN data into useful and functionally meaningful groups. In this study, feed-forward artificial neural network (FANN) and self-organizing maps (SOM) were used as supervised and unsupervised recognition method to understand the global responses to 5-FU. As an unsupervised neural network algorithm, SOM can easily visualize the complex data, which has successfully been used to analyze very large data files in biochemistry fields, including the discovery of gene relationships^30^, classification of complex chemical patterns^31^, and tumor classification^32^. The supervised classification method through FANN yields a compared result. We have investigated whether SOM and FANN can be applied to the analysis of the effects of 5FU on the RN and dRN pools sizes. Our results showed that SOM and FANN could rapidly and reliably cluster RN and dRN pool-size data into different groups. Moreover, feature maps of SOM revealed which RN and dRN pool size was influential for the model and how the RN and dRN pool sizes correlate with each other. This study has attempted to investigate some of the similarities and differences in the performance of these two artificial neural network technologies through the complex data of RN and dRN pool sizes.

## Results

### Cell viability

**Figure 1:**
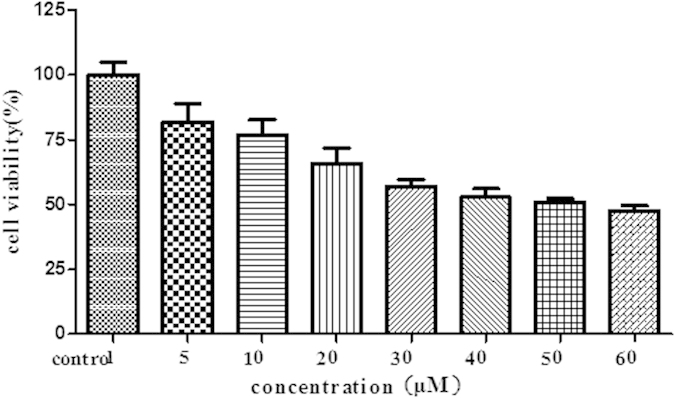
HegG2 MTT assay of 5-FU.

Cell viability was examined by MTT Assay as previously described^33^. HepG2 cells were incubated with various concentration of 5-FU (5, 10, 20, 30, 40, 50 or 60 umol/L) for 72 h. Results of the MTT assay are presented in Fig. 1. IC_50_ was calculated by GraphPad Prism 5 software. The IC_50_ of 5-FU was 49.9 μmol/L.

### Discrimination of samples using FANN

**Figure 2:**
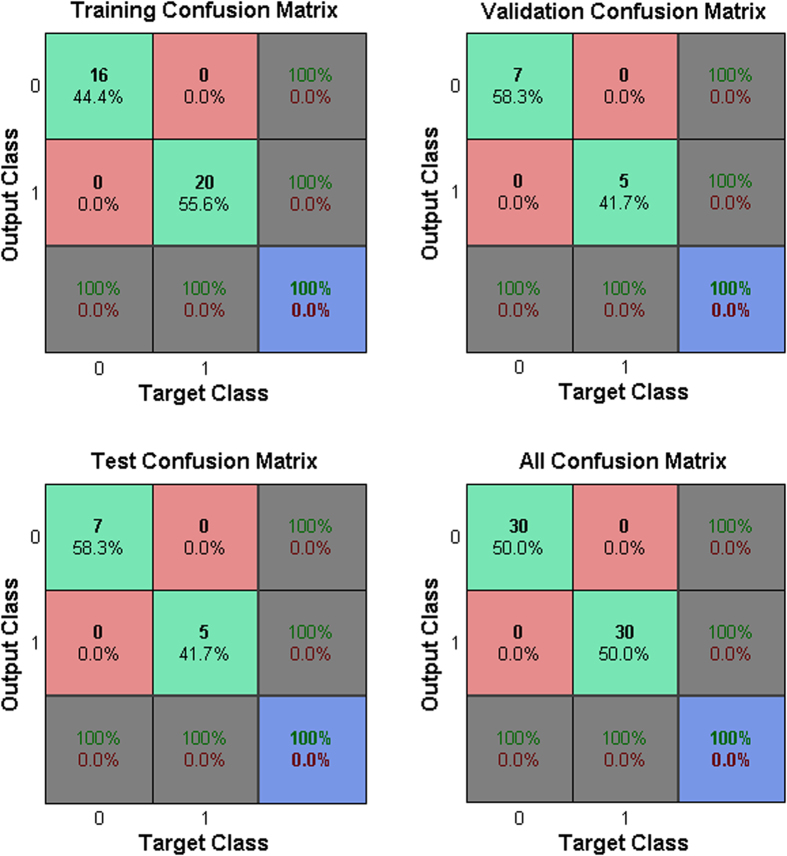
HegG2 FANN results for 60 cell samples before and after 5-FU using concentrations of 27 ribonucleotide and deoxyribonucleotide Pools sizes as input variables.

The RN and dRN pool sizes were analyzed by FANN with a view of establishing whether the separation between control group and 5-FU group was significant by prediction of classes. Totally 60 samples were divided into three groups: 36 samples (60% of samples) for the training data set, and 12 samples (20%) each for the testing data set and validation data set. Figure 2 illustrates the rates of successful prediction was 100%, 100% and 100% for training, testing and validation data, respectively. Totally the rate of successful prediction was 100%, which shows that all samples can be classified correctly by this method according the impact of 5-FU. This result indicates that 5-FU incubation is associated with changes in RN and dRN pool sizes. The procedure summarizes the total alteration in the data and classifies RN and dRN pool sizes by the shape of the time-dependent changing pattern and not by the absolute value of the degree of change.

### Discrimination of samples using SOM

**Figure 3:**
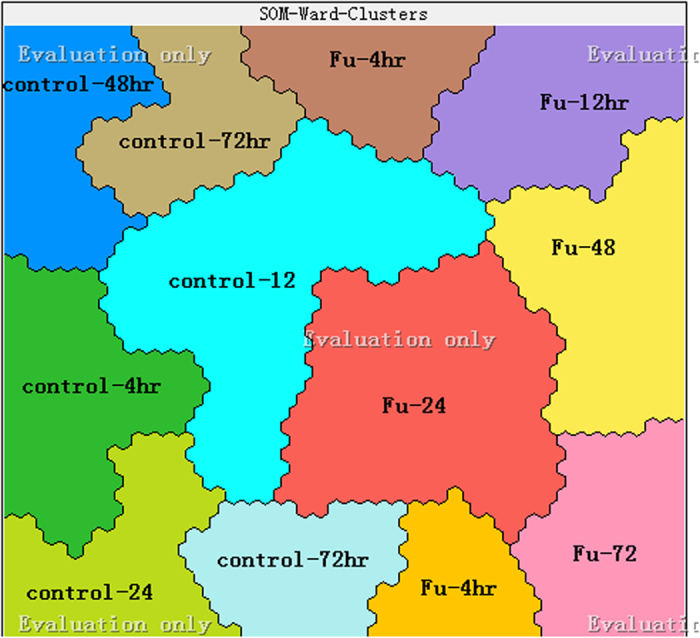
Self-organizing map (SOM) analysis revealing an effect of -5FU on the RN and dRN pool sizes.

In order to assess the effects induced by 5-FU on RN and dRN pool sizes, SOM was used to process data sets. It can visualize the difference in global RN and dRN pool sizes. Thus SOM was useful to explain the mechanism of 5-FU. The SOM described by basic Kohonen algorithm consisted of two-dimensional feature space with lattice neurons. Initially, random values were chosen for the weight vectors *W*_*j*_. The winning neuron I(X) was usually selected by minimizing the distances 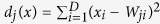. The update formula for weight vector was 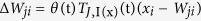 where 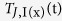 was a Gaussian neighborhood and *θ*(t) was the learning rate. This process was repeated until the node with closet weight vector was found to discretize presentation of high-dimensional input data. By the sophisticated form of multivariate analyses of SOM, the metabolites could be classified into cells on a two-dimensional grid. Different profiles between the cell production and 5-FU incubation were observed in the control group and the 5-FU group. As shown in Fig. 3, 12 clusters of the map could be classified, each representing a group sharing similar characteristics. Control samples at 72 h and samples at 4 h of 5-FU incubation were divided into two sub-groups because of poor reproducibility arising from difference on the initial state of cell in duplication. Generally, the samples from the control group and 5-FU group were located on the left side and right side, respectively.

### Effect of 5-FU on adenosine and deoxyadenosine pool sizes

We used a simple and sensitive LC/MS/MS method to assess alterations in intracellular pool sizes before and after incubation with 5-FU. SOM summarizes the total alteration in the data. Through feature planes of SOM, thus further quantification analyses of RN and dRN pool sizes could reveal which RN and dRN pool size is influential for the model and how they correlated with each other. Feature maps show the distribution of values of the respective input component over the map in different color. The color scale of blue, green and red was used for low values, mid-range values and high values, respectively.

**Figure 4:**
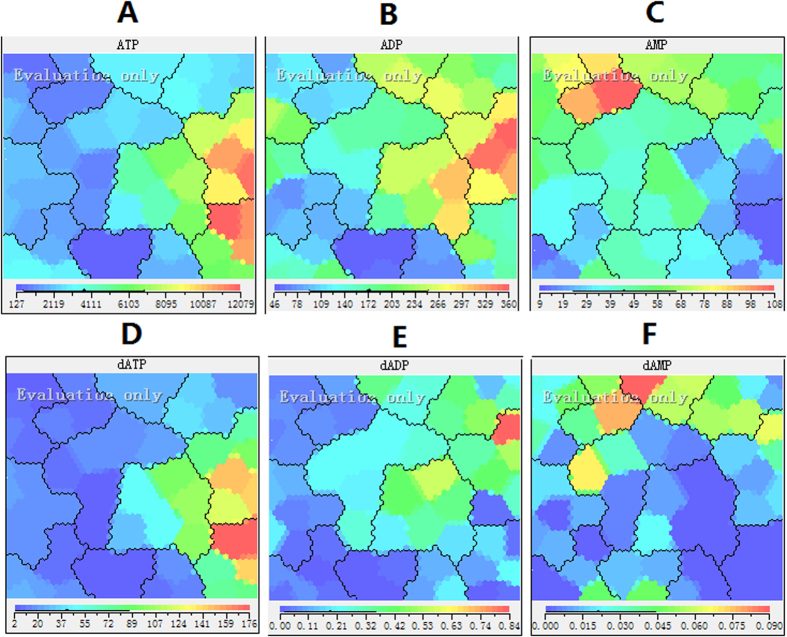
Feature maps of adenosine and deoxyadenosine pool sizes as input attributes. (**A**) ATP; (**B**)ADP; (**C**)AMP; (**E**) dATP; (**F**) dADP; (**F**) dAMP.

As expected, RN levels in HepG2 cells were significantly greater than that of dRN levels (Tables 1, 2). Adenosine ribonucleotides constituted the highest portion of the RN pool. In the beginning, there was no significant difference in RN levels with or without 5-FU incubation. After 12 h of 5-FU incubation, the amount of ATP increased from about 1.5 to 15 fold. The most dramatic increase (about 15-fold) occurred after 72 h of 5-FU incubation. It can be seen form Fig. 4A that the extreme values represented in red in the ATP feature map in two groups after 48 and 72 h of 5-FU incubation.

**Figure 5:**
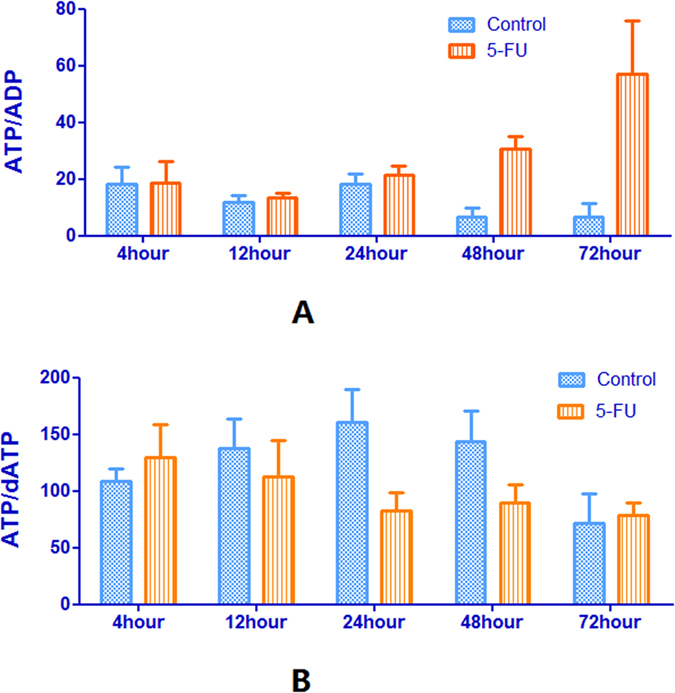
Ratio of ATP/ADP (A) ATP/dATP (B) before and after incubation of HepG2 cells with 50 μM of 5-FU. Each data point is an average of two independent experiments (done in triplicate) and is reported as mean ± standard deviation.

It appears that the increase in the ATP/ADP ratio was exclusively brought about by an increase in ATP concentration and not by a decrease in the ADP concentration (Tables 1 and 3, Fig. 5A). In fact, the amounts of adenosine-monophosphate (AMP) and ADP increased after incubation of 5-FU (Fig. 4B,C). 5-FU could incorporate into all species of RNA, which is an important element of its cytotoxicity. Thus the inhibition of RNA synthesis may be responsible for accumulation of adenosine pool size. The dATP pattern was very similar to that of ATP because of interference with DNA synthesis and incomplete DNA repair caused by the TTP depletion (Fig. 4D). It can be seen from Fig. 5B that the ratios of ATP/dATP were similar before and after incubation of 5-FU. Concomitant dATP accumulation could accentuate the deoxyribonucleotide imbalance and pronounced inhibition of DNA synthesis. At the same time, deoxyadenosine diphosphate (dADP) and deoxyadenosine monophosphate (dAMP) appears to be the most constant during the incubation of 5-FU (Fig. 4E,F).

### Effect of 5-FU incubation on uridine and deoxyuridine pool sizes

**Figure 6:**
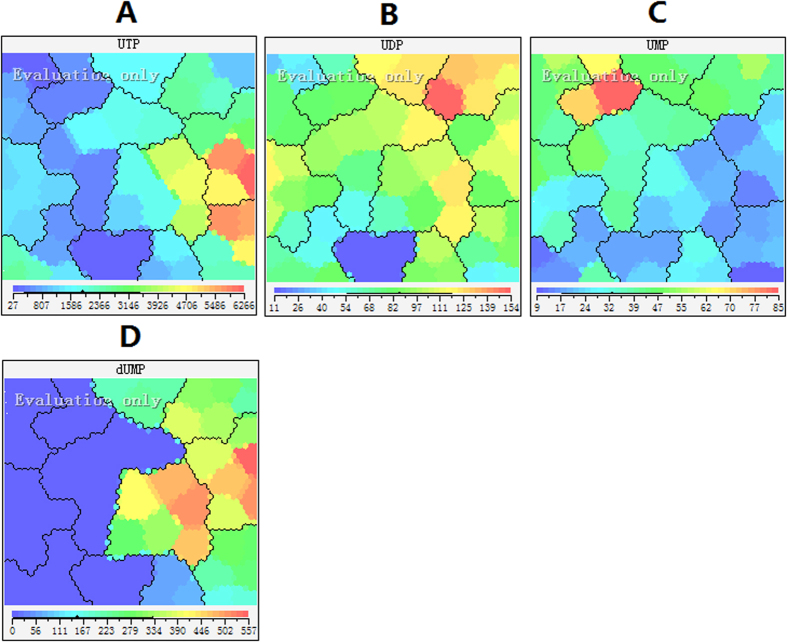
Feature maps of uridine and deoxyuridine pool sizes as input attributes. (**A**) UTP; (**B**) UDP; (**C**) UMP; (**E**) dUMP.

**Figure 7:**
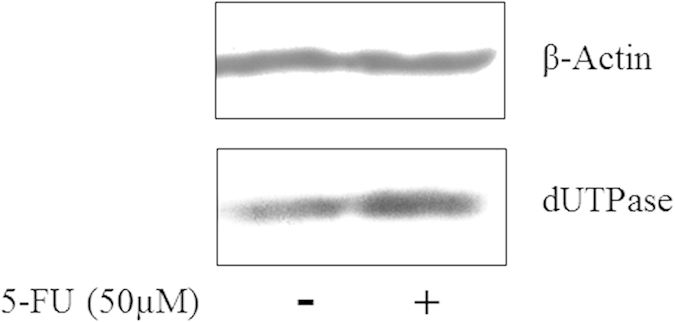
Western blot analysis of the effect of 5-FU on expression of dUTPase.

The uridine pools size exhibited similar behavior as that observed in the adenosine pool size after incubation with 5-FU. Uridine triphosphate (UTP) showed a similar increase (almost 15-fold) as observed with ATP due to the inhibition of RNA synthesis (Fig. 6A). At the same time, evenly distributed colors means there was no remarkable change in the uridine diphosphate (UDP) and uridine monophosphate (UMP) levels with or without 5-FU (Fig. 6B,C). 5-FU acts in several ways, but principally as a TS inhibitor. Interrupting the action of this enzyme blocks synthesis of the pyrimidine thymidine, which is a nucleoside required for DNA replication. TS methylates dUMP to TMP. As expected, dUMP increased after 5-FU incubation from 4 to 72 h (Fig. 6D). It is interesting that the accumulation of dUMP could not subsequently lead to increased levels of dUTP and dUDP. To further investigate these observations, we determined the expression of dUTP nucleotide hydrolase (dUTPase) using the western blot and ELISA. This enzyme catalyses the hydrolysis of dUTP formed to dUMP. The expression of dUTPase was significantly increased after 72 hour incubation with 5-FU (Fig. 7). At the same time, ELISA showed the similar results as western blot analysis (control group: 11.2 ± 1.4 pg/ml versus 5-Fu group: 20.6 ± 2.3 pg/ml, P = 0.004). The amount of dUTPase may play an important role in the tumor resistance to 5-FU. A previous study has demonstrated that the expression dUTPase was associated with the metastatic potential of colorectal cancer and response to 5-FU^34^. The low dUTPase was related with longer overall survival, longer time to progression and better efficacy of 5-FU^35^.

### Effect of 5-FU incubation on thymidine pool size

**Figure 8:**
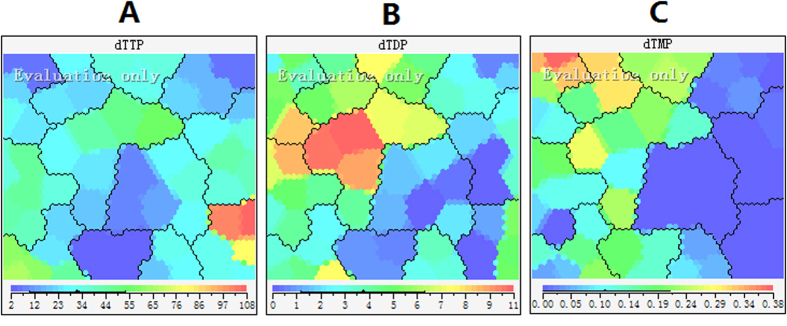
Feature maps of *thymidine* pool sizes as input attributes. (**A**) dTTP; (**B**) dTDP; (**C**) dTMP.

**Figure 9:**
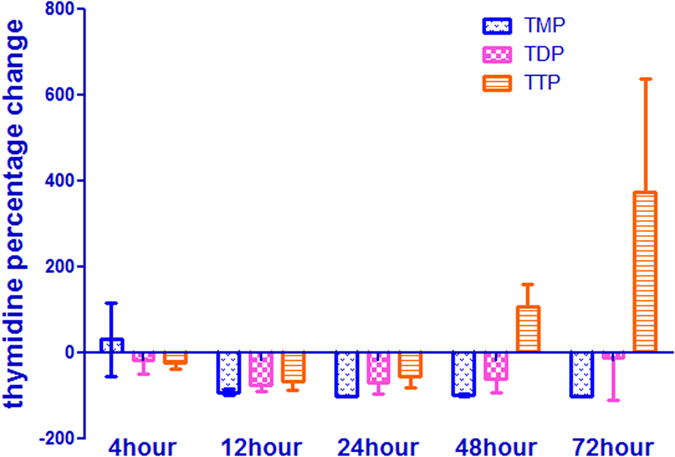
Percent change in thymidine pool after incubation of HepG2 cells with 50 μM of 5-FU. Each data point is an average of two independent experiments (done in triplicate) and is reported as mean ± standard deviation.

In HepG2 cells, TTP was the most abundant dNTP. dATP was the next most abundant, followed by dGTP and dCTP. Although TTP showed the expected decrease (about 2 fold) within 24 h of 5-FU incubation, there was a progressive increase in TTP from 48 to 72 h (Fig. 8A). Additionally, TDP and TMP concentrations decreased with time contrary to that observed with the dUMP pool sizes from 4 to 72 h (Figs 8B,C and 9). dNTP are synthesized via two pathways: the *de novo* pathway and the salvage pathway^5^,^36^,^37^. TS is a key enzyme in the synthesis of pyrimidine in the *de novo* pathway of DNA synthesis and a major target of 5-FU. Although 5-FU still inhibited TS after 48 hours because the concentration of dUMP was increasing and the concentration of TMP was still lower, the cell will begin to use the thymidine for direct synthesis of TTP. Whether or not a thymidine salvage pathway contributes to clinical resistance to 5-FU is difficult to determine with absolute certainty. However, this work confirms that salvage pathway maybe useful for cell self-protection and resistance to 5-FU.

### Effect of 5-FU incubation on cytidine and deoxycytidine pool sizes

**Figure 10:**
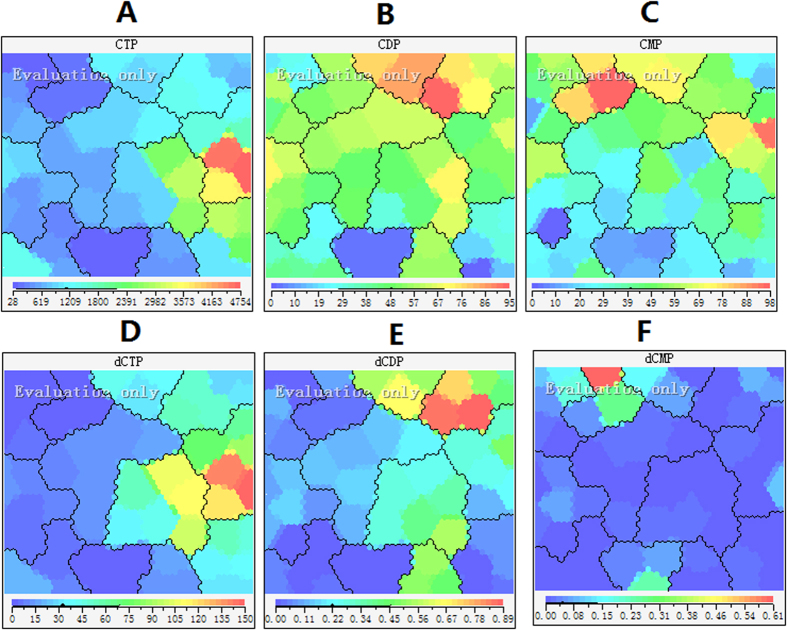
Feature maps of cytidine and deoxycytidine pool sizes as input attributes. (**A**) CTP; (**B**) CDP; (**C**) CMP; (**E**) dCTP; (**F**) dCDP; (**F**) dCMP.

**Figure 11:**
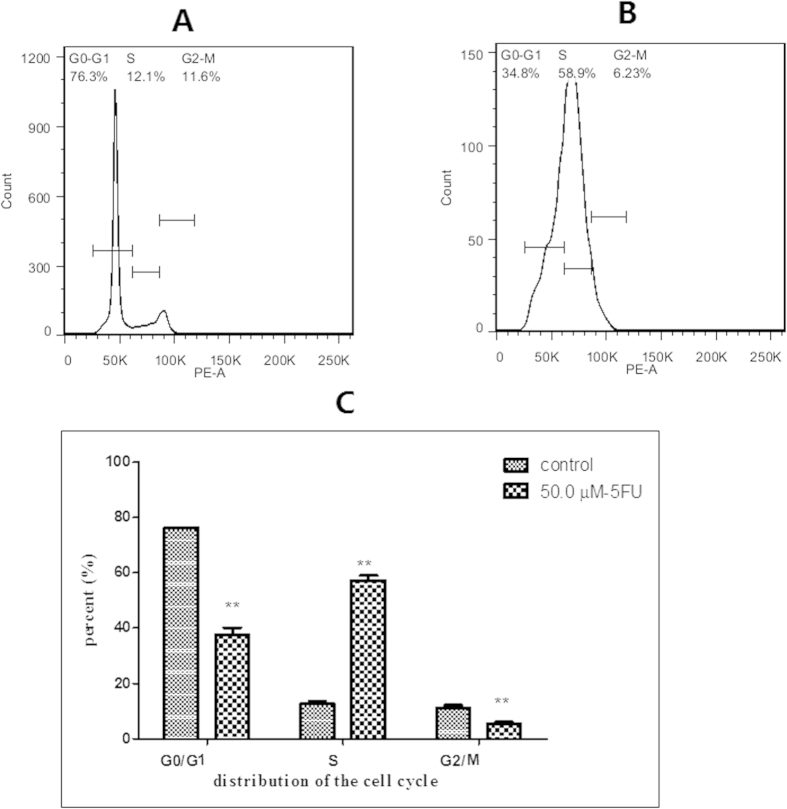
5-FU induced cell cycle arrest at G2/M phase in HepG2. (**A**) Control group; (**B**) 5-FU group; (**C**) The percentages of HepG2 cells at different phases.

**Figure 12:**
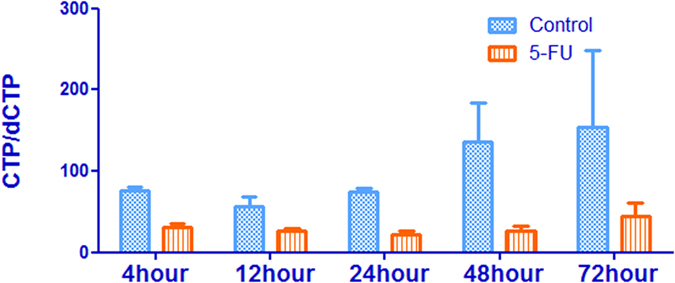
Ratio of CTP/dCTP before and after incubation of HepG2 cells with 50 μM of 5-FU. Each data point is an average of two independent experiments (done in triplicate) and is reported as mean ± standard deviation.

There was a progressive increase in cytidine triphosphate (CTP) concentration after 5-FU incubation up to 72 h, by then the increase reached about 15-fold, which is similar to the trend observed with adenosine and uridine pool sizes (Fig. 10A). Cytidine monophosphate (CMP) and cytidine diphosphate (CDP) remained relatively constant through the duration of incubation (Fig. 10B,C). Flow cytometric analysis in Fig. 11 showed that the population of cells in the G2 phase was decreased by 5-FU at 72 h (5.3 ± 0.7% of cells in the G2 phase of the cell cycle in 50 μM of 5-FU compared with 11.2 ± 0.7% of control cells). Thus 5-FU incubation resulted in an increase in G1/S phase cells, which could lead to increase the ribonucleotides reductase activity. Since TTP is known to inhibit CDP reductase, the decreased TTP caused by incubation of 5-FU may be, at least in part, responsible for this disinhibition of CDP reductase. Consequently, the accumulation of dCTP was caused by the increased CDP reductase activity combined with reduce DNA synthesis (Fig. 10D). Thus there was a decrease in CTP/dCTP ratio in contrast to the increased ratio of ATP/dATP (Table 3 and Fig. 12). The increase in dCTP may be an important contributor to the toxicity of 5-FU because dCTP could active the deoxycytidylate deaminase (dCMP deaminase), which would disturbed the cellular metabolism. At the same time, dCMP and deoxycytidine diphosphate (dCDP) have no significant change in a time dependent manner (Fig. 10E,F).

### Effect of 5-FU on guanosine and deoxyguanosine pool sizes

**Figure 13:**
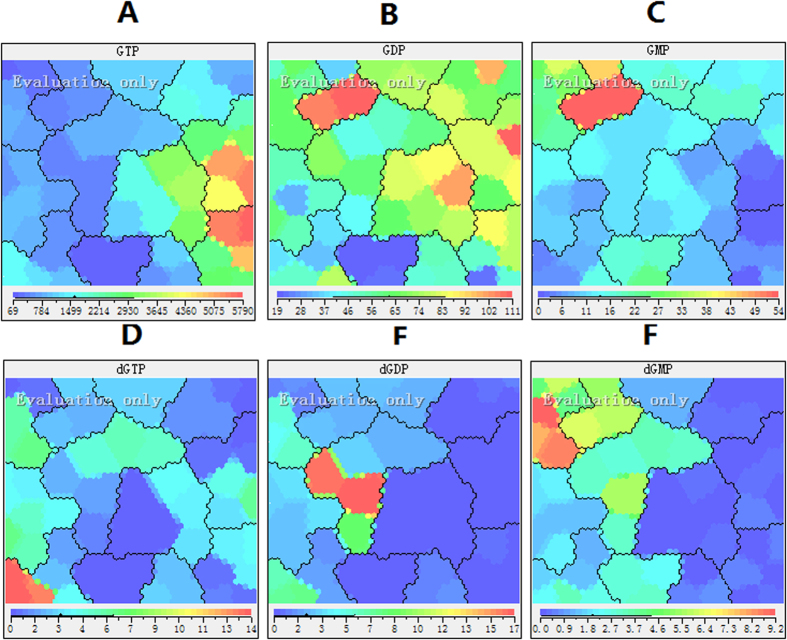
Feature maps of guanosine and deoxyguanosine pool sizes as input attributes. (**A**) GTP; (**B**) GDP; (**C**) GMP; (**E**) dGTP; (**F**) dGDP; (**F**) dGMP.

**Figure 14:**
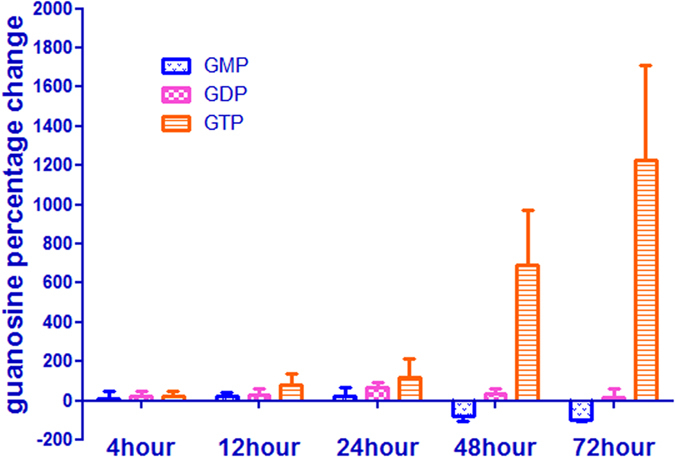
Percent change in guanosine pool after incubation of HepG2 cells with 50 μM of 5-FU. Each data point is an average of two independent experiments (done in triplicate) and is reported as mean ± standard deviation.

Deoxyguanosine pool size decreased after incubation with 5-FU from 4 to 72 h, because the decrease in TTP also represents the reduction of guanosine diphosphate (GDP), hence the deoxyguanosine pool level decreased according to the regulation mechanism of dNTP synthesis (Fig. 13D—F). There were increases in GTP and GDP concentrations, which are consistent with changes in ATP as a result of inhibition of RNA synthesis. (Fig. 13A,B). Thus the ratio of GTP/dGTP after incubation with 5-FU was approximately 2-3-fold greater than that of the control cells without 5-FU incubation. However, the large decrease in the GMP was contradictory to our expectation after 24 h exposure to 5-FU (Figs 13C and 14). The reduction of guanosine monophosphate (GMP) may be useful for cell self-protection and resistance to 5-FU since the antitumor activity of was significantly enhanced by combination of guanosine, and slightly by adenosine, but not by cytidine or uridine^38^,^39^. In additions, guanosine also effected the action of 5-FU, but adenosine, uridine, and cytidine did not. The decreased GMP induced that GDP would be preferentially incorporated in the important enzyme nucleoside diphosphate phosphohydrolase, which is responsible for the conversion of GDP to GMP. Because UDP was also the substrate of nucleoside diphosphate phosphohydrolase, pyrimidine metabolism enzymes would takes precedence over 5-FU in order to maintenance of the correct cellular levels of UMP. This will lead to the decrease of active metabolites of 5-FU.

Finally, compared with dRN pool size, the pattern of NTP pool size showed a similar increase after incubation of 5-FU, which was brought by the inhibition of RNA synthesis and feedback of their metabolism enzymes. A disturbance of the balance of RN and dRN pools in response to chemotherapy may have far-reaching consequences on the activity of many nucleoside analogues. Our data have provided further molecular explanations to support the observed clinical result.

## Discussion

Measurement of all RN and dRN pool sizes in the cell line has been difficult until recently because of the insensitivity of the available LC/MS/MS assay methods. Our paper should be the first report on which individual levels of 27 RN and dRN have been analyzed simultaneously after exposure to 5-FU. In particular, our study elucidates potential differential responses of all RN and dRN pool sizes to different durations of incubation of HepG2 cells with 5-FU.

The changes in deoxyribonucleoside triphosphate pools following exposure to 5-FU are comparable to previously published reports^22^,^27^,^28^,^29^. The changes of dUTP seen in the presence of 5-FU, however, differ in that the increase occured in the deoxyuridine triphosphate only. The lower limit of quantification of dUTP in our assay was about 0.95 pmol/10^6^ cells. The level of dUTP was under detection limit of the assay even if the concentration of dUMP was more than 400 pmol/10^6^ cells. The dUTPase is the key enzyme of dUTP pools and evidence suggest that dUTPase may be catalyzing the degradation of dUTP to dUMP quickly^29^,^40^. These possibilities are under investigation. The amount of dUTP could be associated with response, cytotoxicity resistance and overall survival to 5-FU. Inhibitors of thymidylate metabolism (i.e., the fluoropyrimidines and antifolates) represent an important class of antineoplastic agents^41^,^42^,^43^. Thus our findings could be useful in understanding the mechanisms of TS inhibitors.

The rapid decrease of TTP within 24 hour is a direct result of the inhibitory effects of 5-FU on *de novo* thymidylate. Interestingly, it is not attributable to a progressive decrease in the TTP pool, since after the initial fall in the intracellular concentration of this nucleotide, TTP level began to recover and increase from 48 hour exposure to 5-FU although TDP and TMP continued to decrease compared with control group. This condition therefore confirms that cells started to synthesis the TTP using *salvage* pathway. As a preliminary interpretation of this process it is likely that thymidine kinase activity is enhanced as a result of the loss of feedback inhibition resulting from the lowered TTP pool. Support for the circumvention of the *de novo* pathways of purine and pyrimidine biosynthesis by the salvage pathways comes from our observation that there are sufficient levels of nucleotides to overcome the inhibitory and cytotoxic effects of 5-FU.

Compared with dRN pool size, the pattern of RN pool size showed a similar increase after incubation of 5-FU, which was brought by the inhibition of RNA synthesis and feedback of their metabolism enzymes. A disturbance of the balance of RN and dRN pools in response to chemotherapy may have far-reaching consequences on the activity of many nucleoside analogues. Thus our data have provided further molecular explanations to support the observed clinical result.

In summary, we were able to quantify and investigate the effect of 5-FU incubation on all dRN and RN pools. The observed alterations in pool sizes were consistent with the present understanding of ribonucleotide and deoxyribonucleotide metabolism. We shall continue to investigate the dynamics of interaction between endogenous dRN and the respective metabolites of nucleoside analogues used for cancer chemotherapy and HIV chemotherapy. This work could enhance our understanding of how these analogues work and may predict nucleoside analog efficacy and toxicity.

## Methods

### Chemicals and reagents

LC-MS grade methanol, acetonitrile and acetic acid were purchased from Anaqua Chemical Supply Co., Houston, TX, USA. Hexylamine (HA), diethylamine (DEA), trioctylamine, 1, 1, 2-trichlorotrifluoroethane, stable isotope labeled adenosine-^13^C_10_,^15^N_5_-triphosphate (ATP^13^C,^15^N), dimethyl sulfoxide (DMSO), trypsin-EDTA solution and 3-[(4, 5)-dimethylthiazol-2-yl]-2, 5-diphenyl tetrazolium bromide (MTT) were purchased from Sigma Aldrich Chemical Co., St. Louis, MO, USA. Ultra-pure water was obtained from a Milli-Q Gradient Water System (Millipore Corp., Bedford, MA, USA). For culturing cells, phosphate buffered saline (PBS), Dulbecco’s Modified Eagle Medium (DMEM), penicillin–streptomycin solution and fetal bovine serum (FBS) were obtained from Gibco Invitrogen Corp., Carlsbad, CA, USA. Human hepatocellular cancer cell line (HepG2) was bought from American Type Culture Collection (ATCC), Rockville, MD, USA.

### LC/MS/MS Assay

This was performed on a Thermo Fisher TSQ LC–MS/MS system consisted of an Accela Autosampler, an Accela pump and a Quantum Access triple quadrupole mass spectrometer (Thermo Fisher Scientific Co., San Jose, CA, USA). Data acquisition was performed with the Xcalibur software version 2.0.7, and data processing using the Thermo LCquan 2.5.6 data analysis program (Thermo Fischer). The chromatographic separation was achieved using an XTerra-MS C_18_ column (150 mm × 2.1 mm i.d., 3.5 μm, Waters Corp., Milford, MA, USA). The two eluents were: (A) 5 mM HA–0.5% DEA in water, pH adjusted to 10 with acetic acid; and (B) 50% acetonitrile in water. The mobile phase consisted of linear gradient of A and B: 0–15 min, 100–80% A (v/v); 15–35 min, 80–70% A; 35–45 min, 70–45% A; 45–46 min, 45–0% A; 46–50 min, 0–0% A; 51–70 min, 100–100% A. The liquid flow-rate was set at 0.3 mL/min, and the column temperature was maintained at 35 °C. For all RN and dRN, the following optimized parameters were obtained. The sheath gas pressure reached 40 psi. The ionspray voltage was set at 3000 V for negative mode and 4000 V for positive mode, respectively and the temperature at 350 °C. The Auxiliary gas pressure was 15 psi. Quantification was performed using multiple reactions monitoring (MRM) as previously published^18^.

### Cell Culture

Cells were cultured in DMEM medium supplemented with 10% dialyzed fetal bovine serum (dFBS), 100 units/mL penicillin, 100 μg/mL streptomycin in a 37 °C humidified incubator with a 5% CO_2_ atmosphere. HepG2 cells were seeded in 100 mm by 20 mm dishes (Corning Inc, Corning, NY, USA). After overnight culture, cells were divided into two groups: control group and experimental group. Cells of experimental group were incubated with 50 μM (ID_50_) of 5-FU for different time periods (4, 12, 24, 48, or 72 h). An extra dish of cell line was incubated for cell counting on the day of cell harvest for normalization of nucleotide pools, and the viability assessed by trypan blue exclusion assay (only cells with more than 95% viability were assessed). Control cells were incubated in medium only.

### MTT assay

The inhibitory effect of 5-FU on HepG2 was determined by the cytotoxic MTT assay. HepG2 cells were seeded in 96 wells plate (LabServ, Thermo Fisher Scientific Co., Beijing, China) at 1 × 10^4^ cells/well. After incubation, they were treated with the 5-FU at different concentrations for 72 h. MTT solution (final concentration of 0.5 mg/ml in medium) was added to each well and incubated further for 4 h. The medium was removed, and 100 μl of DMSO was added to each well to dissolve the purple crystals of formazan. Absorbance was measured at 570 nm with a microplate UV/VIS spectrophotometer (Infinite M200 PRO, Tecan Austria GmbH 5082, Grödig, Austria); reference wavelength was 650 nm. The cell number was determined using a hemocytometer. IC_50_ (half maximal (50%) inhibitory concentration) values of 5-FU were calculated by GraphPad Prism software. Cell viability (%) = OD _treated_/OD_control (untreated)_ × 100.

### Preparation of cell pellets

Monolayer cells HepG2 cells were washed with ice-cold PBS once and were trypsinized with 0.25% trypsin-EDTA. Cells from two or three dishes were then re-suspended with 12 mL ice-cold phosphate buffered isotonic saline solution (PBS). After centrifugation at 1,000 rpm for 5 minutes, cell pellet was washed with 1 mL ice-cold PBS again and spun down at 1,000 rpm for 5 min. The cell pellet was incubated with 150 μL of 15% trichloroacetic acid (TCA) containing 7.5 μL of 20.0 uM ATP^13^C, ^15^N as internal standard and placed on ice for 10 min. After centrifugation at 13,500 rpm for 15 min in the cold room, the acidic supernatant was separated and neutralized twice with 80 μL mixture of trioctylamine and 1, 1, 2-trichlorotrifluoroethane (a volume ratio of 45 to 55). Samples were stored at −80 °C until analysis, which was performed within two days.

### Feed-forward artificial neural network (FANN)

Absolute amount of each ribonucleotide and deoxyribonucleotide was used to obtain a data matrix consisting of 60 objects and 27 variables. In this study, RN and dRN pool sizes has been classified using the most common artificial neural network, the two layer feed-forward network with sigmoid hidden and output neurons. The data included the input (RN and dRN pool sizes) and output (with or without 5-FU) that were applied to the input layer and output layer, correspondingly, to make the FANN learn the complex relationship between input and output. Samples were grouped by a 3:1:1 ratio to generate training, testing, and validation samples, respectively. The numbers of hidden neurons were ten. Based on a trial process, the training cycle was repeated until the performance gradient falls below 1.0E^−6^. FANN was performed in the Matlab environment (Mathworks Inc., Natick, MA, USA), running on PC with Intel (R) Core™ i5 CPU 3.2 GHz and 4.0GB RAM.

### Classification based on self-organizing maps (SOM)

Although SOM is a type of artificial neural network, it is a common unsupervised recognition method, which is different from other artificial neural networks. Since SOM uses a neighborhood function to preserve the topological properties of the input space, the distribution of samples on this two-dimensional graph may reveal a pattern that might be correlated to the general characteristics of samples. The Viscovery SOMine 6 package (viscovery.net) has been used to cluster the 60 samples based on the 27 variables. The number of nodes was settled at 2000. Tension was 0.5. The other part kept default parameters.

### Cell cycle analysis

Cells were seeded at 2 × 10^4^ cells/well in 6-well culture plates in duplicate and incubated with 5-FU at 50 μM for 72 h. They were then harvested and fixed in 70% (v/v) cold ethanol overnight at 4 °C. The fixed cells were collected by centrifugation and resuspended in PI/RNase Staining Buffer (Cell Cycle Detection Kit, Nanjing KeyGen biotech Co, Ltd., Nanjin, China) to stain for DNA before final analysis on a FACSAriaTM III Flow Cytometer, Beckon Dickinson and Company, San Jose, CA, USA.

### Preparation of cell extracts and measurement of dUTPase

Cells were washed with ice-cold PBS twice and lysed in RIPA buffer (Cell Signaling Technologies Inc. Beverly, MA, USA). The lysate was cleared by centrifugation at 12,000 × g. dUTPase measured using human deoxyuridine triphosphatase ELISA Kit (USA TSZ biological Trade Co., Ltd., North Brunswick, NJ, USA) according to the manufacturer’s instructions using a microplate reader (Infinite M200 PRO, Tecan uatria GmbH, Grödig, Auatria) at 450 nm.

### Western Blot analysis

Every group cells were harvested and lysed in RIPA buffer (Cell Signaling Technologies Inc. Beverly, MA, USA). Bradford reagent (Bio-Rad, Hercules, CA, USA) was used to determine protein concentration. Then, adding 5ХSDS-loading buffer to give final concentration and heating the tubes at 100 degree C with locked capping for 5 min. The cell lysates (70 μg) were subjected to 12% SDS-PAGE. After electrophoresis, the cell extracts from SDS-PAGE were transferred to nitrocellulose membrane. Then, the membranes were incubated with rabbit dUTPase antibody (Trust Specialty Zeal, TST biological Trade Co., Ltd. USA) and β-actin antibody (Santa CruzBiotechnology, CA, USA)overnight at 4 °C. Furthermore, the membranes were incubated with HRP-conjugated antibodies for one hour. Visualization of the protein bands by using the enhanced chemiluminescence reagents (Invitrogen, Paisley, Scotland, UK). The bands analyzed by using the Image J 1.46r software (National Institutes of Health, Bethesda, MD, USA).

## Additional Information

**How to cite this article**: Guo, J. *et al.* Application of artificial neural network to investigate the effects of 5-fluorouracil on ribonucleotides and deoxyribonucleotides in HepG2 cells. *Sci. Rep.* **5**, 16861; doi: 10.1038/srep16861 (2015).

## Figures and Tables

**Table 1:** Levels of RN in HepG2 cell line before and after incubation with 5-FU at different time (pmol/10^6^cell).

	4hr		12hr		24hr		48hr		72hr	
	**Control**	**5-FU**	**Control**	**5-FU**	**Control**	**5-FU**	**Control**	**5-FU**	**Control**	**5-FU**
ATP	1961 ± 245	2488 ± 554*	1758 ± 614	2734 ± 321**	2582 ± 785	5465 ± 1523**	1085 ± 619	9464 ± 1430**	593 ± 466	8905 ± 2447**
ADP	119 ± 45	161 ± 84	146 ± 28	208 ± 38**	139 ± 33	252 ± 45**	154 ± 55	309 ± 36**	76 ± 27	162 ± 31**
AMP	42 ± 9	52 ± 18	45 ± 4	56 ± 7**	26 ± 13	37 ± 14	66 ± 11	35 ± 18**	70 ± 27	17 ± 8**
GTP	833 ± 143	1026 ± 216	665 ± 261	1188 ± 364*	1110 ± 429	2451 ± 938**	482 ± 283	3817 ± 1240**	306 ± 228	4065 ± 1345**
GDP	50 ± 9	62 ± 12	55 ± 10	73 ± 13*	48 ± 15	80 ± 12**	60 ± 6	83 ± 14**	52 ± 40	60 ± 23
GMP	11 ± 3	12 ± 4	12 ± 1	15 ± 3*	8 ± 4	10 ± 3	27 ± 5	7 ± 5**	35 ± 16	3 ± 3**
CTP	659 ± 229	742 ± 278	787 ± 251	1058 ± 283	953 ± 481	1794 ± 1000	334 ± 217	2921 ± 1452**	123 ± 94	1901 ± 856**
CDP	56 ± 10	70 ± 13	57 ± 7	72 ± 12*	34 ± 7	53 ± 14*	38 ± 15	52 ± 7	29 ± 26	20 ± 11
CMP	35 ± 22	46 ± 24	37 ± 13	38 ± 16	26 ± 8	35 ± 12	45 ± 17	63 ± 19	48 ± 37	32 ± 13
UTP	1268 ± 80	1406 ± 310	1136 ± 699	1770 ± 686	1628 ± 782	2908 ± 1320*	477 ± 321	4220 ± 1463**	253 ± 230	3796 ± 1582**
UDP	94 ± 20	107 ± 15	89 ± 22	129 ± 13**	66 ± 14	99 ± 22**	59 ± 19	101 ± 13**	44 ± 33	74 ± 21
UMP	35 ± 9	36 ± 9	40 ± 5	45 ± 5	20 ± 9	22 ± 5	47 ± 6	20 ± 4**	46 ± 28	16 ± 5*

Note: Each data point is an average of three independent experiments (each performed in duplicate) and is reported as mean ± standard deviation values. (*P < 0.05, **P < 0.01, compared with the control group).

**Table 2:** Levels of dRN in HepG2 cell line before and after incubation with 5-FU at different time (pmol/10^6^cell).

	4hr		12hr		24hr		48hr		72hr	
	Control	5-FU	Control	5-FU	Control	5-FU	Control	5-FU	Control	5-FU
dATP	17.97 ± 1.53	20.63 ± 7.44	13.81 ± 6.54	26.33 ± 9*	17.26 ± 7.28	71.46 ± 30.31**	7.29 ± 3.61	110.42 ± 31.18**	6.99 ± 4.14	118.95 ± 46.35**
dADP	0.1 ± 0.06	0.22 ± 0.09*	0.23 ± 0.02	0.31 ± 0.11	0.04 ± 0.02	0.34 ± 0.13**	0.05 ± 0.01	0.4 ± 0.25**	0.04 ± 0.03	0.09 ± 0.08
dAMP	0.01 ± 0.01	0.04 ± 0.03	0.02 ± 0.02	0.03 ± 0.02	0.01 ± 0.02	0.01 ± 0.01	0.03 ± 0.01	0.03 ± 0.02	0.04 ± 0.02	UDL*
dTTP	40.53 ± 4.37	31.05 ± 5.39**	36.42 ± 13.58	11.92 ± 6.1**	46.62 ± 18.44	20.53 ± 11.05*	16.12 ± 10.26	33.43 ± 7.44**	13.62 ± 11.59	64.4 ± 32.98**
dTDP	5.58 ± 1.97	4.7 ± 1.62	8.18 ± 2.15	2.12 ± 1.1**	4.21 ± 1.66	1.33 ± 0.86**	5.21 ± 1.9	2.03 ± 1.46**	3.07 ± 2.39	2.92 ± 2.56
dTMP	0.11 ± 0.07	0.15 ± 0.09	0.19 ± 0.06	0.02 ± 0.01**	0.12 ± 0.06	UDL*	0.24 ± 0.11	0.0048 ± 0.01**	0.2 ± 0.08	UDL*
dGTP	4.54 ± 0.9	2.69 ± 0.2**	3.26 ± 1.63	0.89 ± 0.58**	8.25 ± 5.55	1.92 ± 1.56*	3.17 ± 2.6	2.51 ± 2.1	1.39 ± 1.03	2.2 ± 1.41
dGDP	3.15 ± 0.36	1.73 ± 0.64**	8.18 ± 6.23	0.35 ± 0.36**	4.28 ± 2.55	0.12 ± 0.11**	3.06 ± 2	0.07 ± 0.04**	1.29 ± 1.25	0.19 ± 0.19*
dGMP	1.8 ± 0.29	0.85 ± 0.4**	3.56 ± 0.96	0.18 ± 0.12**	2.13 ± 0.71	0.15 ± 0.16**	6.71 ± 2	0.24 ± 0.12**	4.29 ± 1.56	0.33 ± 0.23**
dCTP	8.53 ± 2.61	26.12 ± 13.16**	13.69 ± 2.29	40.62 ± 11.8**	12.97 ± 6.76	74.67 ± 30.89**	2.35 ± 1.4	105.32 ± 33.01**	0.64 ± 0.4	40.43 ± 8.32**
dCDP	0.1 ± 0.05	0.54 ± 0.07**	0.15 ± 0.03	0.63 ± 0.22**	0.03 ± 0.02	0.35 ± 0.1**	0.05 ± 0.04	0.28 ± 0.11**	0.03 ± 0.03	0.05 ± 0.03
dCMP	0.03 ± 0.02	0.04 ± 0.06	0.01 ± 0.01	0.03 ± 0.03	UDL*	0.01 ± 0.01	0.09 ± 0.07	0.02 ± 0.04*	0.21 ± 0.2	0.01 ± 0.01*
dUTP	UDL*	UDL*	UDL*	UDL*	UDL*	UDL*	UDL*	UDL*	UDL*	UDL*
dUDP	0.44 ± 0.37	2.98 ± 1.69**	0.6 ± 0.51	2.34 ± 2.25	0.53 ± 0.74	1.39 ± 1.38	0.07 ± 0.08	0.65 ± 0.6*	UDL*	UDL*
dUMP	0.13 ± 0.12	139.86 ± 76.51**	0.2 ± 0.25	298.21 ± 55.11**	0.18 ± 0.15	412.06 ± 84.37**	0.12 ± 0.05	436.19 ± 80.23**	0.09 ± 0.1	230.02 ± 47.87**

Note: Each data point is an average of three independent experiments (each performed in duplicate) and is reported as mean ± standard deviation values. (*P < 0.05, **P < 0.01, compared with the control group); *UDL, under detected limit of assay.

**Table 3:** General properties of HepG2 cell line before and after incubation with 5-FU at different times.

	4hr		12hr		24hr		48hr		72hr	
	**Control**	**5-FU**	**Control**	**5-FU**	**Control**	**5-FU**	**Control**	**5-FU**	**Control**	**5-FU**
ATP/ADP	18.32 ± 5.45	18.86 ± 6.84	11.8 ± 2.42	13.38 ± 1.5	18.32 ± 3.22	21.34 ± 3.05	6.79 ± 2.81	30.73 ± 3.89**	6.7 ± 4.47	56.99 ± 17.26**
ATP/dATP	109.07 ± 9.94	129.63 ± 26.73	137.88 ± 23.83	113.07 ± 28.79	160.48 ± 27.06	82.63 ± 14.83**	143.29 ± 24.91	89.52 ± 14.31**	71.34 ± 24.02	78.71 ± 10.42
GTP/dGTP	186.08 ± 30.51	381.68 ± 81.07**	223.87 ± 55.77	2105.81 ± 1323.45**	187.76 ± 81.16	2562.35 ± 1644.1**	247.4 ± 118.36	2982.16 ± 1844.2**	216.21 ± 20.46	2507.64 ± 1087.48**
CTP/dCTP	76.29 ± 4.39	30.65 ± 4.48**	56.32 ± 10.45	26.35 ± 2.77**	74.84 ± 4.34	22.35 ± 4.27**	135.94 ± 44.17	26.08 ± 5.75**	180.08 ± 80.33	45.13 ± 14.07**

Note: Each data point is an average of three independent experiments (each performed in duplicate) and is reported as mean ± standard deviation values. (*P < 0.05, **P < 0.01, compared with the control group).
